# The Use of the Statistical Entropy in Some New Approaches for the Description of Biosystems

**DOI:** 10.3390/e24020172

**Published:** 2022-01-24

**Authors:** Vladimir V. Aristov, Anatoly S. Buchelnikov, Yury D. Nechipurenko

**Affiliations:** 1 Dorodnicyn Computing Centre, Federal Research Center “Computer Science and Control” of Russian Academy of Sciences, Vavilova Str. 40, 119333 Moscow, Russia; 2 Laboratory of Molecular and Cellular Biophysics, Sevastopol State University, Universitetskaya Str. 33, 299053 Sevastopol, Russia; tolybas@rambler.ru; 3 Laboratory of DNA–Protein Recognition, Engelhardt Institute of Molecular Biology of Russian Academy of Sciences, Vavilova Str. 32, 119991 Moscow, Russia; nech99@mail.ru

**Keywords:** biological systems, statistical entropy, cooperative interactions, macromolecule-ligand binding, adsorption model, kinetic model of metabolism, dissipative structures, Schrödinger’s concepts, negentropy feeding

## Abstract

Some problems of describing biological systems with the use of entropy as a measure of the complexity of these systems are considered. Entropy is studied both for the organism as a whole and for its parts down to the molecular level. Correlation of actions of various parts of the whole organism, intercellular interactions and control, as well as cooperativity on the microlevel lead to a more complex structure and lower statistical entropy. For a multicellular organism, entropy is much lower than entropy for the same mass of a colony of unicellular organisms. Cooperativity always reduces the entropy of the system; a simple example of ligand binding to a macromolecule carrying two reaction centers shows how entropy is consistent with the ambiguity of the result in the Bernoulli test scheme. Particular attention is paid to the qualitative and quantitative relationship between the entropy of the system and the cooperativity of ligand binding to macromolecules. A kinetic model of metabolism. corresponding to Schrödinger’s concept of the maintenance biosystems by “negentropy feeding”, is proposed. This model allows calculating the nonequilibrium local entropy and comparing it with the local equilibrium entropy inherent in non-living matter.

## 1. Introduction

The concept of entropy plays an important role in describing complex processes including thermodynamics, statistics, communications, etc. (for review, see ref. [1]). Some problems of an adequate description of biological structures at microscopic and macroscopic scales are related to entropy.

There is extensive literature dealing with theoretical problems of life phenomena, e.g., see ref. [2]. Some current approaches are presented in ref. [3]. The authors note that: “Although knowledge of biological systems has evolved exponentially in recent decades, it is surprising to realize that the very definition of Life continues to present theoretical challenges.” For the purposes of this article, we can mention some trends in the description of biological organisms as open systems that overlap to a greater or lesser degree with kinetic approaches. For example, work [4] clarifies some of the views that were discussed in the 20th century. The first one is related to E. Bauer, the second to L. von Bertalanffy. Bauer discussed the “stable nonequilibrium” and intended to prove the specificity of the biological sciences against physics; he postulated the need to formulate specific laws of motion that are valid for living matter itself. We assume that ordinary physics is sufficient for describing biological processes. Von Bertalanffy developed the organismic-system theory, in which the process dynamics is inherent inside this system.

It is useful to note other works [5,6,7,8,9]. In ref. [5] the entropy production in the dissipation and irreversibility processes for the open systems is studied. The authors of ref. [6] studied the problem of reconstruction of gene interaction networks after discussing the significance of the notion of entropy in the description of biological systems. Then they considered extensions and potential limitations of the maximum entropy approach. Caro et al. examined the influence of conformational entropy in protein interactions [7]. Demirel studied the use of information (and entropy) notions in living systems and the influence on fluctuations [8]. General physical ideas combined with the modern biological methods are considered in detail in ref. [9].

To consider the biological system as a whole, we develop a kinetic approach, see refs. [10,11], which describes this structure as a nonequilibrium open system. In doing so, biological systems are considered in accordance with Schrödinger’s idea that a biological organism “feeds on negentropy”. Kinetic theory is the basis for at least a qualitative confirmation of this viewpoint. Our approach can be associated with the ideas of von Bertalanffy and Bauer, who introduced the concept of unstable equilibrium related to the characteristics of biological systems. But Bauer, for instance, did not consider a known physical apparatus and appealed to a hypothetical unknown matter and tool.

Schrödinger wrote in his book [12]: “What an organism feeds upon is negative entropy. Or, to put it less paradoxically, the essential thing in metabolism is that the organism succeeds in freeing itself from all entropy it cannot help producing while it is alive.” But Schrödinger proposed his idea without a concrete model. We intend to determine the complexity of biological structures as open nonequilibrium systems.

We can also cite the following judgements of Volkenstein from ref. [13], which in different senses support our approach: “A stationary state is possible only in an open system; such a state might be termed a “flowing equilibrium” … at the heart of all biological phenomena we find the physics of open systems far from equilibrium”. This fuzzy assumption (to return to Bauer’s words) can be related to the kinetic model of the “flowing nonequilibrium” because states far from equilibrium are maintained by two factors: the flow (blood, water, etc.), which transfers the nonequilibrium distribution from input to output, and the reactions of different interactions in this open system.

Some works devoted to various aspects of the concept of entropy as applied to biological systems should be mentioned. Lucia and Grisolia showed that cells are able to convert only part of the energy absorbed [14]. They assert that life is an organizational process, i.e., result of system cooperation between components, with an interconnection between subsystems and super-systems, such that for survival the super-system must export equal or more entropy products than its sub-systems produces, towards maximum conversion of available exergy sources to entropy products. Some thermodynamic models applied to living systems address the investigations for the difference between input and output energy and entropy fluxes (see ref. [15]). Here, the analysis of irreversibility related to this wasted heat can represent a new approach to study the behavior of the cells. Some general proposition has been formulated in ref. [16], where new methods of the bioengineering thermodynamics of a cell are discussed. This allows us to consider the living systems as black boxes and analyze only the inflows and outflows and their changes in relation to the modification of the environment. The authors of ref. [17] attempted to describe systematically the rate of entropy production associated with irreversible processes. They apply the model to the most interesting and relevant case of metabolic network, the glucose catabolism in normal and cancer cells. They expect that their method could potentially be a support for cancer detection. Various issues regarding entropy transformation, in particular those related to lactic fermentation and respiration, are discussed in ref. [18]. The rate of entropy model for irreversible processes in living systems is computed. This model is applied to glucose metabolism. The authors deal with phenomena under conditions of local equilibrium inside and outside a typical cell. Glucose catabolism for normal and cancer cells has been considered and the comparison of thermodynamic ratios between the corresponding entropy rates. The same authors discussed some issues regarding entropy generation and its correlation with heat transfer in cell biology with special concern for glucose catabolism representing the prototype of irreversible reactions and a crucial metabolic pathway in stem cells and cancer stem cells [19]. The questions regarding two thermodynamic principles of the minimum energy dissipation and fastest descent are considered in ref. [20], where decreasing specific entropy production in different biological processes is discussed. It is important to emphasize that all relationships in this and in the other mentioned papers are based on the methods of the nonequilibrium thermodynamics with assumption of the local equilibrium. The important recent paper is ref. [21]. Here, the nonequilibrium description can be as an adequate apparatus. Protein folding should be modeled as it occurs in vivo, that is, in a nonequilibrium, active, energy-dependent process. This can and should stimulate a dedicated program of theoretical, modeling and experimental studies. Schrödinger’s ideas are discussed by Kauffman [22], in particular the need to introduce new laws; we believe that the kinetic method can adequately describe biological systems.

In contrast to and in accordance with the concepts of the mentioned works, we intend to explain some properties of life on the basis of notions of nonequilibrium local states, not in a phenomenological sense but in the rigorous physical model as the first, general physical view of a biological system is explored. From our point of view, statistical entropy is able to reflect a high level of correlation between different parts of a biosystem. The traditional method based on the classical definition of entropy (with local thermodynamic equilibrium) overestimates the entropy of the entire system. Indeed, if the number of microstates for independent parts of the biosystem is calculated in this way, the result will not differ from a similar value for a sample, e.g., a piece of granite of the same mass. But in a real biosystem, its different parts, in particular different organs, depend on each other. In this case, the total entropy is much less than in the first mentioned case.

The purpose of this paper is to define and apply an appropriate measure of the complexity of a biological structure using various entropy concepts. Kinetic, statistical, and thermodynamic theories are the basis for an adequate description of complex entropy transformations.

From the macroscopic point of view, the situation of a real biological system can be reproduced in two (actually related) ways of calculating entropy. The first involves an extended definition of entropy and takes into account correlations in the behavior of parts of the biosystem. The other uses a kinetic equation to describe nonequilibrium states. Entropy of cooperative systems can also be considered.

For Boltzmann entropy, the total entropy of a system can be calculated as the sum of the entropies of nonequilibrium parts of the system. Some generalizations of the concept of entropy will be reviewed and analyzed in order to introduce them into an adequate description of biological systems. In particular, the Kullback–Leibler relative entropy can be used for estimations. This approach can be productive, but it must also describe the dynamics of the biological system over time. A kinetic approach based on model relaxation equations can provide such a model.

The outline of the paper is as follows. In the first part, we considered statistical entropy and its application to macroscopic and microscopic scales, namely, to estimate entropy for the organism as a whole and to transform in cooperative processes for biological molecules with ligand binding to the polymer lattice. In the second part, a simple kinetic model of metabolism allows us to study a biological organism as an open nonequilibrium system maintained by feeding the negentropy (*H*-function in terms of Boltzmann theory). From our point of view the parts of our paper are connected; we consider microscopic and macroscopic limits for entropy and their link through kinetic equation. The statistical combinatorial principle is applied to the biological system (or its subsystems) as a whole, i.e., this is “macroscopic entropy”. Then we calculate the “microscopic statistical entropy” for a simple element of the system. Finally, the statistical entropy is a main part of kinetic description which can connect different levels.

## 2. Entropy and the Role of Correlations of Parts of an Organism as a Whole

First, we want to give precise meaning to the intuitive idea that a biosystem has less entropy than a “non-living” system of the same mass. The traditional formulation of the statistical entropy related to the logarithm of the system’s possible states is given in ref. [23]. The probabilities of each state are equal. The increase in the total entropy after thermal contacts of parts of the system is maintained.

There is an important proposition in ref. [24] that real biological systems can be maintained due to slow relaxation processes in high-molecular-weight compounds. Therefore, we can consider stable biological structures for a scale of the mean relaxation time. The kinetic relaxation model equation is used to describe the nonequilibrium steady systems. This true correlation can be described by a model of the biological organism as a whole, and this apparatus can be provided by the kinetic equation with an appropriate problem statement, but one can estimate the entropy by a simple method.

The traditional thermodynamic approach considers a biological system as a combination of subsystems in equilibrium, so it is natural to estimate entropy as a set of independent parts, with entropy equal to the non-biological object of the same mass (or a colony of unicellular organisms). This view is based on the assumption that the probabilities of all states are equal, but a different formula must be used to estimate entropy. Indeed, we must calculate the value of nonequilibrium statistical entropy. This value will, of course, be smaller than the first one due to the multiple connections between different parts and organs in a biological system. Thus, correlations denote that these parts are not independent, and the probability is less.

Total entropy is not a sum of entropies of different parts of a nonequilibrium system, because the total probability is not the product of the probabilities of the different parts. The probability for the nature piece of the non-living material, say granite, can be calculated as the product of the probabilities of different independent parts. We can exchange parts. But for the living system it is not so: all parts in fact are connected one to the other. To compute total entropy, we can calculate the product of the probabilities of the different parts and then subtract the correlation term between those parts. This term may depend on the manner of dividing the parts on the subsystems.

Let us evaluate the entropy of a biological system in terms of explaining that system as a composition of independent parts and correlated parts. This is incorrect because the parts of an organism are strongly connected. For a colony of one-cell organisms this is the number of all rearrangements, namely (10^13^)!. For a multicell organism, each cell can be unique (for example, in the brain). In this case the number of combinations must be equal to 1. An example of a biological system with unique arrangement of each cell (neuron) is the brain. In this case entropy *S* = ln 1 = 0. A similar estimation was made in ref. [24], but, according to the thermodynamic criterion, it can be proposed that any biological system is almost no more ordered than a piece of rock of the same mass. Thus, the entropy of a set of 10^13^ different one-cell organisms is almost equal to the entropy of the human body containing 10^13^ cells. This strange statement is based on the analogy with the thermodynamic estimate of entropy, for example, when cooling a relatively large amount of water. But building a living organism (related to the mentioned value of entropy) is a very difficult task because an organism is a complex nonequilibrium system (see further the kinetic model of metabolism).

We tried to explain in more detail the basis of our model, and in this section we consider the stationary state of the organism. So here we are not studying organ growth. But combinatorial formulas are used in the traditional way. We distinguish between a piece of inanimate matter, where rearrangements are possible, and living matter, where these operations are in question. This approach is common for various statistics, in our situation corresponding to Boltzmann statistics, where particles can be rearranged at a given level. In any case, a living organism is fundamentally different from inanimate matter, in which parts can be changed without changing the essence of the system. In fact, assuming that it is possible to rearrange the cells of only individual organs, we generalized the result of Blumenfeld [24], who considered the limit when all living cells are unique. But let us emphasize the difference with this position on the ease of obtaining a large value of negentropy by the body in comparison with the thermodynamic process of cooling a small amount of water. Blumenfeld also tried to introduce the not entirely explained term “value of entropy”. But a really large amount of living negentropy can be explained from the statistical point of view applied to nonequilibrium systems. Here we must consider the growth of the organism. “Cooking” a living organism is not like cooling (or heating) a little water. The organism develops from an ovulated cell through the embryonic stage to a complex hierarchical living system. The transition from one level to the next requires a large amount of statistical negentropy, which can be calculated for nonequilibrium states.

The common view (presented, for example, in the mentioned work) that, strictly speaking, the concepts of entropy and thermodynamic probability make sense only for equilibrium states, is wrong. After all, Boltzmann’s theory is not limited to this case. The statistical entropy of kinetic theory can also be applied to nonequilibrium systems.

Thus, for a rough (but correct) estimate, it is possible to divide the organism into organs; in every organ it is possible to exchange cells but for organs it is not possible. The number of combinations in this case will be less than in the case of an independent set of cells. In the first case the number of combinations is (10^13^)! and in the second case ((10^12^)!)^10^ if there are 10 different organs and each contains 10^12^ cells. It is clear that the number of combinations in the second case is less, and therefore entropy is also less.

It is more correct to calculate this number of variants as ((10^12^)!)^10^ for the Boltzmann formalism. Thus, the thermodynamic probability is (10^13^)!/((10^12^)!)^10^. For the simplest example, we consider 100 cells. For a set of 100 cells we have 100!. If we have 10 organs of 10 cells each, the number of variants is (10!)^10^. The thermodynamic probability is 100!/(10!)^10^, and it is greater than one. Note that we use the so-called thermodynamic probability of the macroscopic state. It is equal to the number of ways (the number of microscopic states) in which a given macroscopic state can be realized. This probability is greater than or equal to one. The thermodynamic probability *W* is related to the entropy *S* by *S* = *k_B_* ln *W*, where *k_B_* is Boltzmann constant.

Therefore, in this example, the entropy for the distribution function with “numbers of completeness” equal to 10 is actually constructed. And if we take the value of ln *W* = ln 100!/(10!)^10^ (here a constant *k_B_* is omitted for simplicity), we obtain the well-known expression for the entropy, and one can see that this value for a multicellular biological organism is significantly less than the entropy for a set of different cells.

The entropy calculation for this case is analogous to the classical calculation of the statistical Boltzmann entropy with the total number of particles *N* and the numbers of particles in states *N*_1_, …, *N_m_*. The number of complexes (thermodynamic weight) is equal to *N*!/(*N*_1_! …*N_m_*!). Here m is the number of levels or in terms of biological approach the number of organs.

The final conclusion is as follows: entropy of a multicell biological organism according to statistical approach is less with respect to the matter of the same mass. Therefore, statistical entropy, used even in the model version of Boltzmann statistics, can be used. This leads to the proposal to measure the entropy of the living organism.

## 3. Statistical Entropy in Description of Living Systems at the Molecular Level

For more than a century, equilibrium statistical thermodynamics has been successfully applied to describe biological processes at the molecular level. Physico-chemical experiments in model in vitro systems, where such biological macromolecules as proteins, their complexes, RNA and DNA, as well as small molecules (peptides, dyes, oligonucleotides or proteins etc. to be further referred to as ligands) are present, have shed light on many mechanisms underlying cellular processes. Conventionally, model experiments are performed at room temperature and constant pressure; therefore, Gibbs free energy, Δ*G*, and the related equilibrium association constant, *K*, are used to describe the relevant thermodynamic system. In terms of physics, the process of reversible ligand binding on the macromolecule surface is a physical adsorption phenomenon that belongs to the class of lattice models, where the lattice polymer consists of ligand binding sites [25,26].

Consider a polymer with bound ligands as an open system, i.e., it can exchange substance (ligands) with the solution. Cooperative interactions often occur between bound ligands close to each other on the macromolecule surface. Of particular interest are the so-called allosteric effects in proteins and nucleic acids, when binding of one ligand molecule to a macromolecule causes its rearrangement, i.e., a change in conformation at a certain distance from the bound ligand, so that the binding of subsequent ligand molecules occurs with a different constant. A typical allosteric effect is the interaction of oxygen with the hemoglobin tetramer. Thousands of papers have been devoted to the study of this process, and its quantitation led to the famous Hill equation more than a hundred years ago, then the Adair equation and a dozen other models and equations that are now considered classical (e.g., see refs. [27,28]). Note that the current COVID-19 pandemic makes relevant some works devoted to the influence of medium acidification and the cooperative oxygen binding to hemoglobin (the so-called Verigo–Bohr effect) [29,30,31].

Cooperative effects are manifested in the vast majority of processes occurring at different levels in living organism: from molecules to cells and tissues. Such effects are found when ligands bind to various macromolecules, in particular to receptors, during signal transduction in cell or in the case of intercellular interactions. Although low-molecular-weight ligands do not take part in the processes of protein folding [32,33,34] and DNA melting, the latter are substantially cooperative. Thus, cooperativity significantly affects both intracellular and intercellular regulation of life processes [25,35,36,37,38].

When binding on the macromolecule, ligands can both help each other and hinder binding; the first case corresponds to positive cooperativity, and the second one, to negative cooperativity, or anticooperativity. The authors of ref. [39] investigated negative cooperativity and showed that it leads to ultrasensitivity of the system, which means “the way the system responds to various doses of the ligand can change dramatically from a very gradual one to a switch-like behavior”. Cooperative effects appear in the aggregation of small molecules [40,41,42,43,44,45], which also plays an important role in biomedical applications.

For a rigorous thermodynamic description of cooperative processes, it is necessary to understand how basic state functions behave depending on the parameters of cooperativity. One of the fundamental state functions describing the nature of a system is statistical, or configurational, entropy. The role of mixing entropy in the reactions of intermolecular stacking aggregation was elucidated in ref. [46]. Below we will try to study the behavior of statistical entropy using the example of adsorption of small molecules on a linear polymer and show that calculations of entropy make it possible to shed light on the processes occurring in the system.

## 4. Model of Adsorption and Entropy Evaluation

Consider the following thermodynamic system: macromolecules with *N* equivalent binding sites and the ligand molecules with molar concentration *c*, capable of occupying one binding site are in equilibrium in solution at constant temperature and pressure. Let us denote the equilibrium constant of ligand binding to a single macromolecule site as *K*, and the parameter of cooperativity as *ω*. The cooperativity can be due to contact interactions between ligands occupying adjacent binding sites. Values of *ω* < 1 correspond to negative cooperativity, or anticooperativity, and values of *ω* > 1 correspond to positive cooperativity; when *ω* = 1 the adsorption is non-cooperative [39].

**Figure 1:**
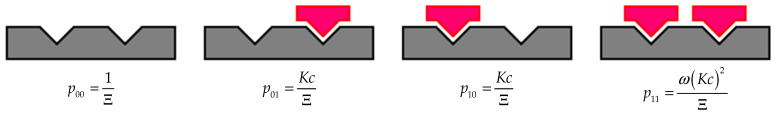
Four possible states of the system consisting of two-site lattice and binding ligands, and their corresponding probabilities (indices 0 and 1 stand for an empty and an occupied site, respectively).

The grand partition function is the quantity that most completely describes the system. There are several ways of constructing a grand partition function for a system in which ligands are adsorbed on the polymer lattice, namely, matrix method based on the Ising model [47], the combinatorial method first used by Scatchard [48], and some others. The grand partition function, Ξ, of a system consisting of ligands and a macromolecule with *N* = 2 binding sites is actually the sum over all 2*^N^* = 4 possible microstates. Taking 1 and *Kc* as the statistical weights of an empty and an occupied site, respectively, one can readily construct the grand partition function going over all the states (see Figure 1) [49]:

(1) $$\Xi =1+2Kc+\omega {\left(Kc\right)}^{2}.$$

Once the grand partition function is known, we can calculate all thermodynamic parameters of the system, in particular entropy.

Let us define the statistical, or configurational, entropy of the system as a measure of the uncertainty arising from the adsorption of ligands on the lattice. Indeed, if it is known that *q* ligands are bound on the lattice, uncertainty arises as to which of the *N* sites are occupied by ligands and which are free. This entropy is due to the number of arrangements in the linear sequence of *q* occupied sites and (*N*–*q*) free sites. It is known from statistical mechanics that such entropy, *S*, per mole of lattices is equal to:

(2) $$S=-R\sum_{i=0}^{1}\sum_{j=0}^{1}p_{ij}\ln p_{ij},$$

where *R* is the gas constant, *p_ij_* is the probability of finding the lattice in which two sites are in the *i*th and *j*th states. The explicit forms of the probabilities are given in Figure 1.

The question arises: how does the entropy of the system behave when the ligand concentration and the cooperativity parameter change? Let us find the explicit form of entropy, *S*, on the basis of Equation (2):

(3) $$S=-R\left(p_{00}\ln p_{00}+p_{01}\ln p_{01}+p_{10}\ln p_{10}+p_{11}\ln p_{11}\right).$$

Substitution of the probabilities from Figure 1 to Equation (3) yields

(4) $$S=R\left[\ln\Xi -\frac{Kc}{\Xi }\left(2\left(1+\omega Kc\right)\log\left(Kc\right)+\omega Kc\ln\omega \right)\right].$$

**Figure 2:**
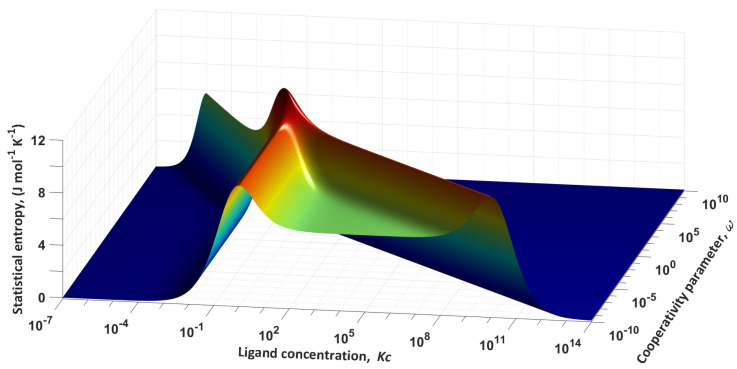
The statistical entropy as a function of two arguments: *S* = *S* (*Kc*, *ω*).

Let us represent entropy *S* as the function of two independent variables *Kc* and *ω*. Generally, the equilibrium constant *K* and the concentration *c* are physically distinguishable quantities, but in our analysis they exist inseparably. Moreover, the constant *K* is the measure of steady-state affinity of ligand to a site of the macromolecule, hence playing the role of an additional coefficient to the concentration. Therefore, we will further use them as a single quantity to be referred to as generalized ligand concentration or simply ligand concentration. Figure 2 shows the corresponding surface.

**Figure 3:**
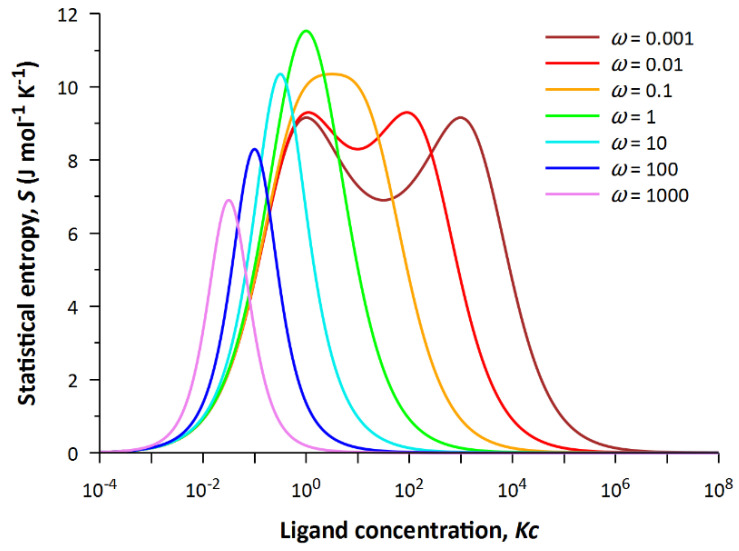
The dependencies of statistical entropy on the product *Kc* at different values of *ω*.

Figure 2 shows that the entropy has an absolute maximum at *Kc* = 1 and *ω* = 1. For a complete analysis of the surface, we construct several cross-sections of the plane at different values of the cooperativity parameter. The set of dependencies *S*(*Kc*) at fixed values of *ω* is shown in Figure 3.

Obviously, the dependence of entropy on generalized ligand concentration turns out to be non-trivial, since it acquires two maxima instead of one at values of the cooperativity parameter *ω* < 1. The two maxima are due to the fact that two states at the same concentration of free ligand in solution have the same statistical weight. One of the maxima always corresponds to *Kc* = 1. When ω≪1, the state with statistical weight *ω*(*Kc*)^2^ = 1 competes with the latter state.

**Figure 4:**
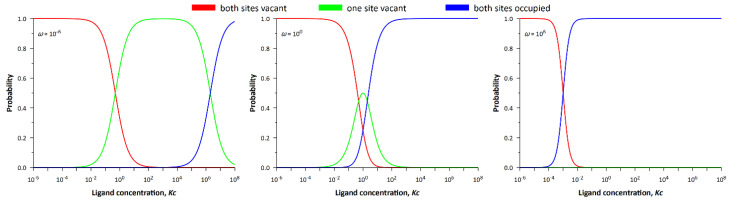
Dependencies of the probability density of the system states on the ligand concentration.

Figure 4 shows the dependencies of the probability density of the system states on *Kc*. If the cooperativity parameter is sufficiently small (ω≪1), the three states successively replace each other. With increasing ligand concentration, the probability of the empty lattice begins to decrease, while the probability of the lattice with occupied and free sites increases. As ligand concentration continues to increase, the probability of a fully occupied lattice increases. In the limit of *ω* → 0, one would expect the latter event to occur at infinitely high ligand concentrations, which is impossible, and this is in perfect agreement with our ideas. Indeed, the existence of two neighboring occupied sites is prohibited in the infinitely anticooperative process (neighbor exclusion model) [50].

This model is the link to lattice model of “solid rod gas”, in which one ligand occupies several sites at once upon binding. Equations of state for such a system were first derived by Tonks [51]. However, the equation for the case of ligand adsorption on the lattice was not known, and it was derived again [52,53].

Cooperative interactions between such ligands were considered by Zasedatelev, Gursky, and co-authors [54,55]. Also worth mentioning is the approach of McGhee and von Hippel [56], which has become widespread among biochemists and molecular biologists. The next step forward in the description of cooperative interactions was the application of the Ising model to describe the situation when ligands can bind to DNA in different orientations (see refs. [57,58]) and to describe the cooperative binding of ligands to microarrays [59]).

In the model described, macromolecules without ligands, with one and two adsorbed ligands (as well as free ligand molecules) are in equilibrium in solution. Let us assume that we are conducting tests according to the generalized Bernoulli scheme: we take a macromolecule out of the solution and look at how many ligands are adsorbed on it, then we return it to the solution and take out the next one. In this case, we have three test outcomes. But in the limit of infinite cooperativity *ω* → ∞, only two types of macromolecules will be observed: without ligands and with two adsorbed ligands at once (see ref. [60]).

**Figure 5:**
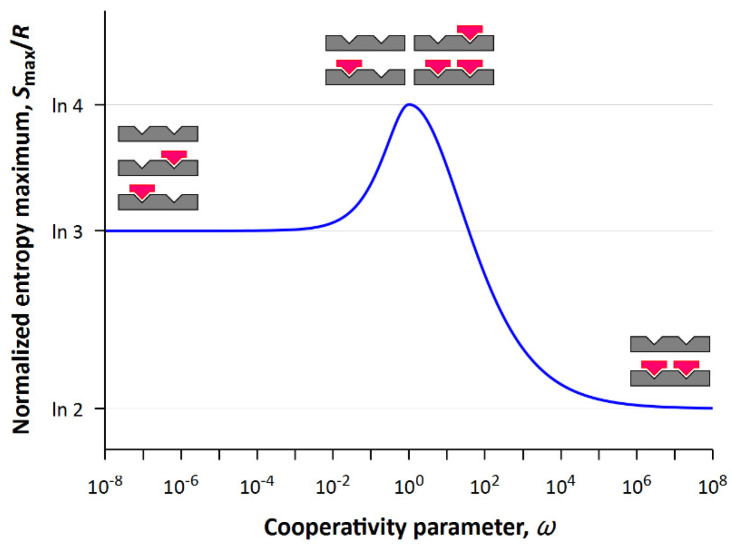
Dependence of maximum entropy on the cooperativity parameter.

From the comparison of Figure 4 and Figure 5 follows an important conclusion that the entropy of the system is maximal at the intersection of probability densities, i.e., when corresponding events are equally probable. Equal probability means that there is no prevailing state of the system and leads to uncertainty, the quantitative measure of which is statistical entropy. In the case of negative or positive cooperativity, the processes of ligand binding depend on each other, whereas in the case of noncooperative binding these processes proceed independently. For this reason, the greatest value of entropy in the maximum (see Figure 4 and Figure 5) is observed precisely for the non-cooperative process (*ω* = 1).

Representing the projection of the two-dimensional surface onto the (*S*, *ω*) plane, one can obtain the dependence of maximum values of entropy on the cooperativity parameter (see Figure 5). Obviously, the maximum of statistical entropy corresponds to the system with non-cooperative interactions (*ω* = 1) in which all four states are equally probable. As the cooperativity parameter (ω≪1) decreases, the state with completely filled lattice becomes less and less probable, and as the cooperativity parameter (ω≫1) increases, the states with a half-filled lattice become less and less probable.

The maximum of molar entropy of the system, *S*_max_, absolutely coincides with the fundamental Boltzmann equation, up to Avogadro constant, *N_A_*:

$$S_{\max}=N_{A}\cdot k_{B}\ln W=R\ln W,$$

where *W* is thermodynamic probability, which bears the similar meaning as the same quantity mentioned above.

Note that in real systems, when ligands bind to receptors, substrates bind to ribosomes, etc., and the ligand binding to the first and second sites can be characterized by different constants [61,62]. When analyzing experimental data, the researcher confronts the so-called inverse problem, i.e., with the reconstruction of model parameters of the binding curves. In this case, uncertainty arises, and the cooperativity parameter can be determined only under specific assumptions. There is also a known problem that the results of calculating the curves in a model with included anticooperative interactions between ligands coincide with the results of calculations in the presence of heterogeneous binding, but not cooperative. To solve this problem, the researcher needs to set up binding kinetics experiments [38].

## 5. Kinetic Model of Metabolism and Entropy

The kinetic method actually uses the *H*-theorem as a sequence of the kinetic theory. The developed simple kinetic model of metabolism [10,11] makes it possible to implement the mentioned idea that a living system feeds on negentropy (not positive energy). This model, based on ref. [11] can relate the size (and age) of biosystems to the intensity of the inner reaction processes.

Another proposal by Volkenstein [13], “…a sort of ‘entropy pump’ is needed to pump entropy out of the open system” is realized by a kinetic model with a negative entropy (or *H*-function) pump, or the *H*-function flux into an open system. In other words, the entropy flux for this stationary system increases from input to output according to the *H*-theorem, and for this nonequilibrium open steady system the energy flux is constant.

We consider a simple model of metabolism with transformation of states of substance on the basis of kinetic theory and the entropy corresponding to it. This investigation (study) can be considered initially on the basis of one-dimensional problems solved by the kinetic methods. The so-called nonuniform relaxation problems (NRP) [10,11,63] have been studied as a simple model for simulation of the biological systems. We begin with the kinetic relaxation model equation (the so-called BGK equation or *t*-model):

(5) $$\xi \frac{\partial f}{\partial x}=\frac{1}{\tau }\left(f_{M}-f\right).$$

Here, *f* = *f* (*x*, *ξ*) is the distribution function, *x* is a physical coordinate, *ξ* is velocity, *τ* is a specific relaxation time, *f_M_* is the Maxwellian (equilibrium distribution). Equation (5) is an equation of the so-called BGK type (see ref. [64]); moreover one can imply that a constant characteristic relaxation time appears in Equation (5). Such an equation is really connected to the Boltzmann transport equation, but it is simpler and can potentially reflect relaxation processes in more complex than gaseous media.

The formulation of the boundary NRP problem is described, e.g., in ref. [11]; the left boundary condition is the following nonequilibrium function. Namely,

$$\begin{array}{ll} f\left(0,\xi \right)=0 & \left(0<\xi <1.5\right),\\ f\left(0,\xi \right)=1 & \left(1.5<\xi <2.5\right),\\ f\left(0,\xi \right)=0 & \left(2.5<\xi <3.5\right),\\ f\left(0,\xi \right)=1 & \left(3.5<\xi <4.5\right),\\ f\left(0,\xi \right)=0 & \left(4.5<\xi \right). \end{array}$$

The distribution function *f* has the usual meaning, namely the number of particles with given velocities for certain quantities of space and time (only translational degrees of freedom are considered here, and to generalize the motion of biological molecules, other degrees of freedom must also be taken into account). The boundary conditions are of a model nature and describe the strong nonequilibrium of the substance entering the body during metabolism.

This upstream nonequilibrium distribution at the point *x* = 0 then changes for larger *x* and then tends to equilibrium downstream. Here we consider a steady spatial nonuniform problem as analogous of the known uniform relaxation problem where *f* tends to equilibrium in time. In our steady system we consider the evolution of *f* in space from the nonequilibrium state inflow to the equilibrium state outflow. The term downstream is traditional; it denotes the points that are down in the flow.

This implies that the kinetic energy of the mean longitudinal velocity of the blood is sufficiently greater than the chaotic energy of particles in blood. Therefore, the back flow, i.e., the part of the distribution function with the negative velocities, can be negligible (it denotes that the elements of the blood move in one direction).

Therefore, there is a small parameter $\alpha =\left(\xi -u_{0}\right)/u_{0}$, where *u*_0_ is the mean boundary velocity for *x* = 0. For the distribution function we construct the analytical solution using a method of expansion in this parameter. For the first order approximation in a small parameter (of the ratio of internal energy to kinetic energy) we obtain

(6) $$f\left(x,\xi \right)=f\left(0,\xi \right)\exp\left(-a\left(\xi \right)x/u_{0}\tau \right)+\left(1-\exp\left(-a\left(\xi \right)x/u_{0}\tau \right)\right)f_{M0}\left(\xi \right),$$

$$f_{M0}\left(x,\xi \right)=n_{0}{\left(\frac{m}{2\pi T_{0}}\right)}^{3/2}\exp\left(-\frac{{\left(\xi -u_{0}\right)}^{2}}{2T_{0}}\right).$$

Here, $a\left(\xi \right)=1-\left(\xi -u_{0}\right)/u_{0}$, macroscopic parameters for the equilibrium distribution downstream *f_M_*_0_, are computed through the macroscopic parameters of the nonequilibrium distribution upstream. The main term in the exponent that determines the spatial relaxation is the same order as in the zeroth approximation. Thus, *l* = *u*_0_*τ*, where *l* is the characteristic spatial value of decay of the nonequilibrium state, *τ* is the average time of interactions (collisions). One can generalize the result if considering *τ_inn_*, i.e., the characteristic mean time of the biochemical reactions, *f_Minn_* = *f_M_*(*x*, *e*) is the equilibrium function with the temperature *T_inn_* of the biosystem (the body temperature). Here the temperature appears in the standard expression for the equilibrium distribution (Maxwellian) *f_Minn_*.

The traditional statistical Boltzmann entropy, used to describe spatial nonequilibrium states, is the following moment:

(7) S=−∫flnfdξ.

Equation (7) can represent the entropy for the strong nonequilibrium distribution, which in fact is described in this chapter and given by the formulas before Equation (6). Really, the kinetic theory by Boltzmann deals with the distributions removed far from equilibrium. Equation (6) shows such distributions on velocity for different physical coordinates of the problem under consideration.

The entropy flux is written according to the ordinary definition adopted in the kinetic theory. The *x*-component of the entropy flux is as follows

(8) $$S_{x}=-\int \xi_{x}f\ln fd\xi .$$

Here *ξ_x_* is the *x*-component of molecular velocity. The entropy flux enters through the left boundary and exits (with a larger value of the entropy flux) through the right boundary of the considered one-dimensional system.

First, we consider the change of entropy flux in space from “nonequilibrium input” to “equilibrium output”. For the steady-state case under consideration, the entropy efflux and the entropy created in the system as a result of interactions are balanced out. From Equation (5), the *H*-theorem for this one-dimensional stationary case implies an increase in the entropy flux, namely (*S_x_*)_downstream_ > (*S_x_*)_upstream_.

**Figure 6:**
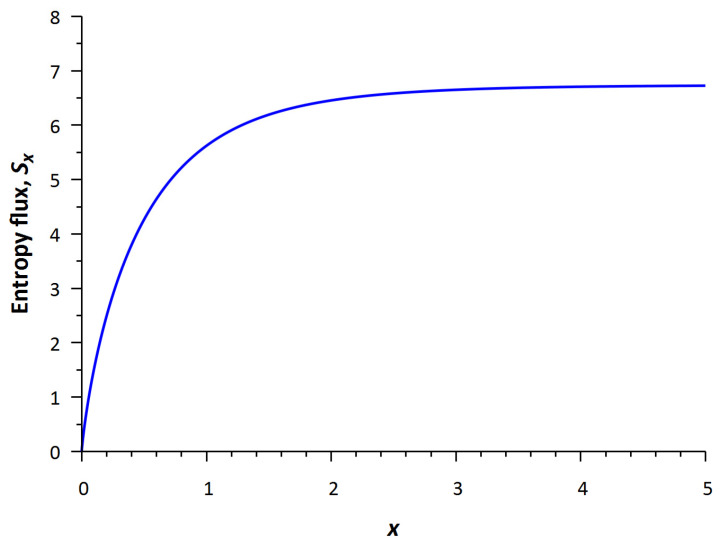
The profile of the entropy flux, *S_x_*.

Figure 6 shows the profile of the increasing entropy flux *S_x_*(*x*) calculated from Equation (8). That is consistent with Schrödinger’s ideas that life is powered by negative entropy (negentropy). Indeed, the *H*-function (*H* = −*S*), i.e., negentropy decreases. This flux is the entropy pump mentioned above. Therefore, the difference of output and input entropy fluxes reads

(9) $$\Delta S_{x}=S_{x}\left(L\right)-S_{x}\left(0\right).$$

It can be denoted as the magnitude of negentropy that feeds on the biological system. Here, *L* is the effective spatial region (associated with the size of the biosystem).

**Figure 7:**
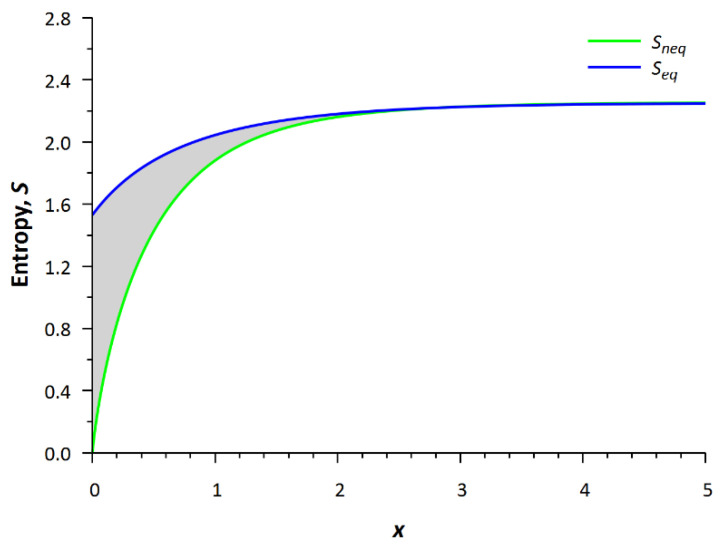
Profiles of nonequilibrium, *S_neq_*, and equilibrium, *S_eq_*, entropies.

The profiles of the equilibrium and nonequilibrium local entropies are shown in Figure 7. The term nonequilibrium entropy means that when calculating the entropy according to Equation (7), the nonequilibrium distribution function is used in the integral, and after substituting the equilibrium function (Maxwellian) into the integral in Equation (7), we obtain, respectively, the equilibrium entropy. The difference between the nonequilibrium (green bottom line, a “life line”) and the equilibrium (blue upper line, a “death line”) entropies with the same density, velocity and temperature is calculated at any local spatial point.

The local nonequilibrium entropy is less than the local equilibrium entropy since this difference is associated with the process of the spatial uniform relaxation. This problem is adequate in the case when the system is instantly closed, e.g., by stopping the flow of blood, then the relaxation process proceeds in the usual way and the distribution tends to equilibrium. Negentropy is understood as the *H*-function (entropy *S* = −*H*), so this quantity is actually the Boltzmann entropy, and it can be calculated for any nonequilibrium state.

It is seen that the local nonequilibrium entropy is less than the local equilibrium entropy. The difference between equilibrium and nonequilibrium total states, taking into account the local nonequilibrium entropy, is calculated as follows (this is the shaded part in Figure 7).

(10) $$\Delta S=\int_{0}^{L}\left(S_{eq}\left(x\right)-S_{neq}\left(x\right)\right)dx.$$

where *S_eq_*(*x*) and *S_neq_*(*x*) are the values of statistical entropy at equilibrium and nonequilibrium states, respectively. This difference, Δ*S*, from Equation (10) can give a possible estimate of the complexity of living objects. This formula actually characterizes the “integral distance” (“metric”) between the “living line” and the “unliving line” in Figure 7, i.e., the complexity of a living organism is a measure of its remoteness from the equilibrium state, which is the simplest basic description of the distribution in terms of velocities or energies.

This value can be called the “life” budget (“life reserve”). Note that the model kinetic equations for mixtures with chemical reactions also demonstrated similar results for one-dimensional NRP, and calculations for two-dimensional NRP confirm this conclusion.

We consider a living system as a structure with entropy less than that in a non-living system of the same mass. Volkenstein’s words [13], “In the case of a living organism, entropy attains its maximum in the equilibrium state—otherwise known as death”, are confirmed by comparison of local nonequilibrium and equilibrium entropies. We can assume that in the closed system the value of Equation (10) is zero. Once the open system is closed, the local equilibrium state will be approached for the time on the order of relaxation time *τ*, i.e., the characteristic time of the basic biochemical reaction. For example, this can be done by stopping the blood flow. Formally, this can be modeled by considering a system with opaque walls at points *x* = 0 and *x* = *L*.

In this case, the right-hand side in Equation (5) is not equal to zero, but the velocities are equal to zero, so it is necessary to introduce the time derivative into the left-hand size, i.e., we have the equation in the form

(11) $$\frac{\partial f}{\partial t}=\frac{1}{\tau }\left(f_{M}-f\right).$$

For this uniform relaxation problem, the initial condition is the distribution function from Equation (8), and the analytical solution analogous to Equation (6) has the following form

(12) $$f\left(t,\xi \right)=f\left(x,\xi \right)\exp\left(-t/\tau \right)+\left(1-\exp\left(-t/\tau \right)\right)f_{M}\left(x,\xi \right),$$

where the local Maxwellian *f_M_* depends on the density *n*(*x*), mean velocity *u*(*x*), and temperature *T*(*x*) calculated using the distribution *f* (*x*, *ξ*) from Equation (8). The relaxation process ends at any point with equilibrium, and the entropy in Figure 7 approaches the upper curve. This line will be unchanged even if the mean velocity is zero. Indeed, we can obtain that the entropy for equilibrium with the same density and temperature and with different mean velocities is the same. For the equilibrium entropy from Equation (7) we have

$$S\left(f_{M}\right)=-\int f_{M}\ln f_{M}d\xi =-\int f_{M}\left(\ln n+\frac{3}{2}\ln m-\frac{3}{2}\ln\left(2\pi kT\right)-\frac{m{\left(\xi -u\right)}^{2}}{2kT}\right)d\xi .$$

A value $\int f_{M}\frac{m{\left(\xi -u\right)}^{2}}{2kT}d\xi =p$, where *p* is pressure that does not depend on the mean velocity. Thus, after stopping the motion the equilibrium entropy will be the same and appeal to the top line. This means, for example, with the cessation of the movement of blood in the cardiovascular structures after the cessation of the work of the heart, this only leads to interactions and spatial uniform relaxation, i.e., tend to “death”. Therefore, relaxation leads to a local equilibrium at each spatial point after several relaxation times and the transition from a “living line” *S_neq_* to a “non-living line” *S_eq_*.

Here we assume that the relaxation time is much shorter than the time of advection between the parts of the spatially nonuniform system with local equilibrium. This process is the next problem. The closeness of the system maintained supported by fast advection processes, such as blood transport, leads to isolation of different parts of the system. Rapid relaxation is realized in each of them. Of course, these parts are not isolated from each other. But these processes of advection such as diffusion will be relatively slow and between parts in local equilibrium. Nonequilibrium thermodynamics does not extract specific properties of living systems, since unlike kinetic theory it is conditioned by equilibrium local states. Then, when the distribution tends to the global equilibrium the process will be relatively slow under the corresponding condition of local equilibrium at any point. Transfer in this non-living system will be due to gradients of different values and will be a diffusion type process. For this case, nonequilibrium thermodynamics will be a correct theory because there is a local thermodynamic equilibrium. Thus, these processes will proceed until a spatial uniform distribution of relaxation in the system is achieved.

## 6. Discussion and Conclusions

In this paper we have considered the possibilities of the concept of entropy as applied to a biological system at different levels. The aim was to study the entropy apparatus for the system as a whole and some of its parts up to the molecular level and to obtain general statements and, possibly, correlations. As a general conclusion for the meanings of our work we can cite the following common words of Volkenstein from ref. [13]: “…entropy has turned from a mere shadow of an omnipotent sovereign to a powerful entity determining the very existence of life on Earth”.

We have tried to discuss the influence on entropy of correlations between the actions of different parts of the whole organism, intercellular interactions and control, as well as cooperativity on the microscopic level.

For a multicellular organism as a whole, Boltzmann’s approach was used to calculate the thermodynamic weight and statistical entropy. In contrast to the opinion of some biophysicists, entropy for a multicellular organism is much less than the entropy for the same mass of “non-living matter” or even a colony of unicellular organisms. Indeed, if each cell in a given organism plays a unique functional role in the system, the cells cannot swap places, and therefore the thermodynamic weight is unity and entropy has a minimum value, that is, zero. For a colony of cells, we can swap any pair of elements of this system, and we will get the maximum value of statistical entropy. In the intermediate case of dividing a system into different organs, we cannot swap these organs without destroying the whole organism, but we can swap the cells of each organ among themselves. Thus, we get an intermediate value of entropy here.

For the “microscopic level” we can conclude that cooperativity always reduces the entropy of the system; a simple example of ligand binding to a macromolecule carrying two reaction centers shows how entropy agrees with the ambiguity of the result in the Bernoulli test scheme.

A similar binding scheme is realized in the case when a macromolecule with two binding centers is DNA on which the repressor protein is adsorbed. In this case, the entropy of binding leads to noise in gene expression, which can be measured (see, for example, ref. [65]). In this case, a change in ligand-repressor concentration leads to a change in cell color, i.e., phenotype. A thermodynamic model that takes into account the cooperativity of ligand binding allows us to establish a connection between the molecular and cellular levels [49]. A similar model is developed in this work; and it allows simulating different modes of cooperative ligand-receptor binding and, consequently, the possibility of switching cellular metabolism.

The simple kinetic model of metabolism proposed earlier allows us to implement Schrödinger’s idea that a living system is powered by negative entropy (negentropy) rather than positive energy; the model is a quantitative expression of this assumption of feeding a biological organism with negentropy (see Figure 6).

For this one-dimensional open nonequilibrium system obeying the relaxation model equation, the energy flux through the boundaries of this system is constant. But the entropy flux increases from input to output. In other words, the negative entropy (negentropy) flux is negative. Here, negentropy is the *H*-function, where *H* = −*S*, and *S* is the nonequilibrium statistical entropy. It is important that this kinetic simplest metabolic model deals with a local nonequilibrium state (unlike, for example, irreversible thermodynamics), and from our point of view, the local nonequilibrium state is a specific property of the living system. This kinetics model allows us to calculate the nonequilibrium local entropy and compare it with the local equilibrium entropy inherent in non-living matter. Integral characteristic of difference between equilibrium and nonequilibrium local entropy can be considered as “life reserve”.

It is also proposed to bring together the macroscopic and microscopic levels of organismal complexity closer together, using, in particular, new definitions of entropy.

Metabolism means the replacement of molecules on the time scale of a biochemical reaction. But the position of body parts changes slowly. Therefore, it is useful to present an appropriate distribution. The entropy of structural distributions in aging biosystems has been determined. Cellular metabolism is required to reduce it. This can lead to a decrease in position entropy. Real cellular metabolism in some living organisms can be an example of resistance to the increase in the entropy of aging. We plan to generalize these models and study the issue in the near future. The so-called Kullback–Leibler relative entropy (Kullback–Leibler divergence) can be useful for this purpose.
